# Supplementary material for: A novel hypergraph model for identifying and prioritizing personalized drivers in cancer
Source: PLoS Comput Biol. 2024 Apr 29;20(4):e1012068. doi: 10.1371/journal.pcbi.1012068 (PMC11081510; doi:10.1371/journal.pcbi.1012068)
Supplement: S1 Text — (DOCX) [file pcbi.1012068.s001.docx]

# S1 Text: Supplementary Material for “A novel hypergraph model for identifying and prioritizing personalized drivers in cancer”

## Comparison evaluation at cohort-level

To compare the personalized-level methods with the cohort-level methods, we applied an adapted version of PageRank by regarding the personalized driver gene ranking score as the voter’s preference for candidates (genes). The aggregated ranking of genes can be derived to represent their priority to be drivers. By way of illustration, we defined a directed graph with all the mutated genes in the cohort as vertices. Let $A$ be adjacency matrix representation of the graph, a 0-1 matrix (if gene $i$ co-mutated with gene $j$ in a sample, then $A\left( i,j \right)=1$). The weights of the directed edge are stored in the matrix $W$, in which $w(i,j)$, the weight of directed edge $e(i,j)$ is defined as the number of votes, obtained by counting how many samples voted for $j$ instead of $i$ by comparation the PDRWH-score in each sample. We calculated the transition matrix of the random walk $P$ as follows:

$P=W^{T}D^{-1}$ (1)

where $D$ is a diagonal matrix composed of the column sums of the matrix $W$. Therefore, the cohort level ranking can be obtained by random walk algorithm as follows:

$\vec{v}_{0}=\frac{1}{n}\vec{I}$ (2)

$\vec{v}_{(t+1)}=\alpha P\vec{v}_{(t)}+(1-\alpha)\vec{v}_{0}, t=N$ (3)

where $n$ is the number of all the mutated genes in the population samples, $\vec{I}\in R^{n}$ is a vector where each element is 1. When there is no longer a significant update in the ranks, the importance of driver genes at cohort level is determined numerically by the stabilized $\vec{v}_{t}$.

For personalized driver gene prediction methods, PersonaDrive, Prodigy, SCS and our method PDRWH, we applied this adapted version of PageRank to aggregated the ranking of genes significance from individual patient samples to a cohort-level. As for DawnRank, we adopted its original predicted driver gene list on cohort-level, because DawnRank designed a modified Condorcet Voting with a penalty heuristic for itself. Using aforementioned general reference driver genes as a benchmark, we generated receiver operating characteristic curve (ROC) and the area under the curve (AUC) to evaluate the true positive and false positive rate.

## In vitro cell-based assays

Our in vitro cell-based assays are divided into three aspects. In the first aspect, we investigated the effect of differential *LRP1* expression between cancer tissues and normal tissues on cell survival. So that we detected the expression of *LRP1* between GES-1 and GC cells, using the expression level of gene *GAPDH* as a reference, as well as the expression of *LRP1* between GC tissues and adjacent tissues by immunohistochemistry. Then the overall survival analysis of *LRP1* was done using Gene Expression Profiling Interactive Analysis (GEPIA, http://gepia.cancer-pku.cn/). The enzyme gene GAPDH is a housekeeping gene, which is expressed at high levels in almost all tissues, and generally has constant protein expression in similar cells or tissues. Therefore, it is widely used as a standardized internal reference for the experimental operations such as the extraction of total RNA, poly A + RNA, Western Blot, etc. In the second aspect, we verified the efficacy of the three siRNAs, si-1, si-2, and si-3, in inhibiting *LRP1* transcription on HGC-27 cells transfected with these siRNAs by Western Blot. In the third aspect, in order to further investigate the effect of *LRP1*’s activity on the function of gastric cancer cell lines, we took measures to perform wound healing assay, cell apoptosis assay, and cell proliferation assay still on HGC-27 cells for both the experimental and control groups. Particularly, the cell cycle profile of control and *LRP1* knockdown cells were also analyzed. Detailed experimental conditions are presented as follows.

### Cell culture

Human gastric cancer (GC) cell lines MKN-28, MKN-28, HGC-27, AGS and SNU-1, which showed *LRP1*-mutation in Broad Institute Cancer Cell Line Encyclopedia (https://portals.broadinstitute.org/ccle), and human normal gastric epithelial cell line GES-1 were obtained from BeNa Culture Collection (Beijing, China). All cells were cultured in RPMI-1640 medium (HyClone, GE healthcare; Chicago, IL, USA), supplemented with 10% fetal bovine serum (FBS) (Biological Industries; Beit-Haemek, Israel), 100 IU/mL penicillin and 100 μg/mL streptomycin (Gibco, Thermo Fisher Scientific; Waltham, MA, USA), at 37°C in an atmosphere of 95% air and 5% CO2.

### Transient transfection with small interfering RNA (siRNA)

HGC cells were transfected for 48 h with synthesized *LRP1* or negative control siRNA (GenePharma, Shanghai, China) using 6-well plates with 100 nmol of siRNA and 5 μL Lipofectamine 2000 (Invitrogen, Carlsbad, CA, USA) according to the manufacturer’s instructions. The sequences of validated siRNA for *LRP1* were:

1. forward 5’-CCAGGUCAGAUGCCAUUUATT and

reverse 5’-UAAAUGGCAUCUGACCUGACCUGGTT for si-1;

1. forward 5’-CCACCUGCAUGAGUUUAATT and

reverse 5’-UUAAACUCAUAGCAGGUGGTT for si-2;

1. forward 5’-GGCCGUGGAUUAUCACAAUTT and

reverse 5’-AUUGUGAUAAUCCACGGCCTT for si-3;

1. the sequences of negative control were

forward 5’-UUCUCCGAACGUGUCACGUTT and

reverse 5’-ACGUGACACGUUCGGAGAATT.

### RNA isolation, real-time RT-PCR

Total RNAs were extracted with TaKaRa MiniBEST Universal RNA Extraction Kit (Takara) followed the manufacturer’s protocol. The primers for *LRP1* were 5’-CTATCGACGCCCCTAAGACTT-3’ and 5’-CATCGCTGGGCCTTACTCT-3’. Quantitative RT-PCR was carried out using the 7500 Real-Time PCR instrument (Thermo Fisher), and β-actin was used as internal control for mRNA assays. Relative expression levels of target gene were quantified by 2-ΔΔCt method from triplicate experiments.

### Wound healing

For the wound healing assay, cells were transfected with siRNA and seeded into 6-well plates at a density of 5x105 cells/well for 24 h. Linear wounds were scratched on the 100% confluent monolayer using a pipette tip. Cells were cultured in medium without FBS and images were captured at 0 and 24 h and analyzed using the Scion image software (version 4.0.3.2; Scion Corporation, Frederick, MD, USA).

### Cell apoptosis assay

Cell apoptosis assay was performed to detect whether *LRP1* regulated the cell apoptosis. Briefly, cells transfected with siRNA were harvested, resuspended and stained using an Annexin V-FITC apoptosis detection kit (BD Biosciences; San Jose, CA) accordinge th manufacturer’s protocol. Then, cells were detected by flow cytometer BD facaria II and the data were analyzed using the CellQuest Pro software (BD Biosciences).

### Cell cycle assay

The cell cycle assay was performed using a cell cycle and apoptosis kit (Beyotime). Briefly, cells transfected with siRNA for 24 h were cultured with serum-free RIPM 1640 medium for 24 h, then, cultured with serum-containing medium for another 24 h. Subsequently, cells were digested with trypsin and fixed for 30 min at 4°C in pre-cooled 70% ethanol. Cells were stained with propidium iodide (PI) and analyzed by a flow cytometry (BD FACSCalibur; BD Biosciences, Franklin Lakes, NJ, USA) for cell cycle analyses.

### Cell proliferation assay

Cell proliferation was determined using an EdU kit (BeyoClick EdU Cell Proliferation Kit with Alexa Fluor 488, Beyotime, China). Briefly, cells transfected with siRNA for 24 h, cells were incubated with EdU for 3 h, fixed with 4% paraformaldehyde for 15 min, and permeated with 0.3% Triton X-100 for another 15 min. The cells were incubated with the Click Reaction Mixture for 30 min at room temperature in a dark place and then incubated with Hoechst 33342 for 10 min.

### Western Blot

Total proteins (30 μg) were separated by SDS-PAGE. Gels were transferred onto PVDF membranes by wet transfer, then, membranes were blocked in 5% BSA in TBST (20mM Tris-HCl, 150 mM NaCl, 0.05% Tween 20, pH8.0) at 37℃ for 2 h, incubated with primary antibody *LRP1* (#abs133272, Absin) and GAPDH (#5174, Cell Signaling Technology) overnight at 4℃, then incubated with appropriate HRP-conjugated secondary antibody, finally, the specific bands were visualized using super sensitive ECL chemiluminescence detection reagent (Tiangen; Beijing, China) and imaged with Chemidoc XRS+ (Bio-Rad Laboratories; Hercules, CA, USA).

### Immunohistochemical analysis

GC tissue microarrays (TMAs, #HStmDis030PT01, Shanghai Outdo Biotech Co.) were used for immunohistochemical analysis of *LRP1*. Briefly, TMAs were dewaxed, and antigen retrieval were performed using citrate buffer. Slides were blocked and incubated overnight with anti-*LRP1* (1:50) antibody, and incubated 1 hour with HRP-conjugated secondary antibodies. 3,3'-diaminobenzidine substrate was used for antibody detection.
