# Supplementary figures and images for: A novel hypergraph model for identifying and prioritizing personalized drivers in cancer
Source: PLoS Comput Biol. 2024 Apr 29;20(4):e1012068. doi: 10.1371/journal.pcbi.1012068 (PMC11081510; doi:10.1371/journal.pcbi.1012068)

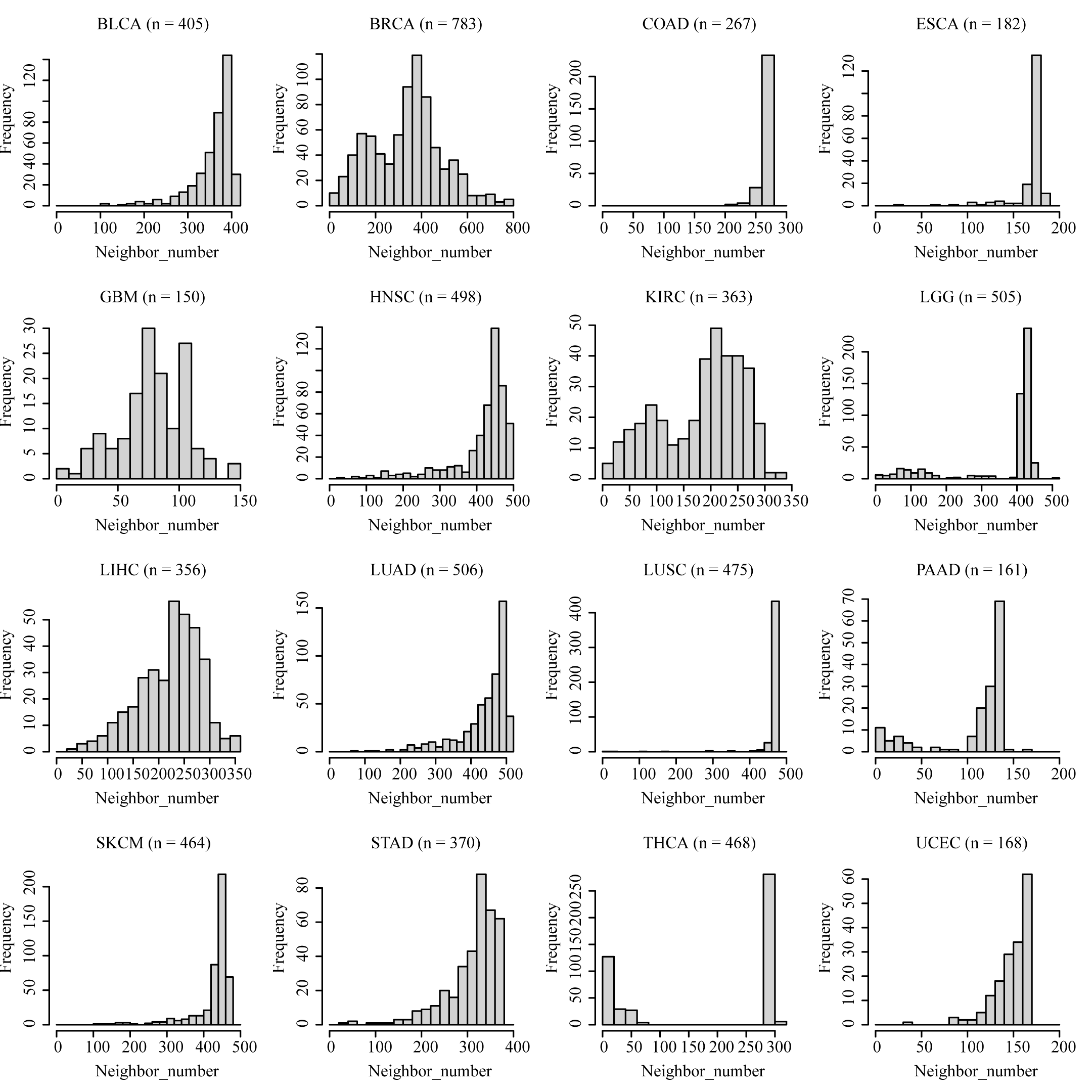

Supplement: S1 Fig — The n in parentheses represents the number of tumor samples. (TIF) [file pcbi.1012068.s002.tif]

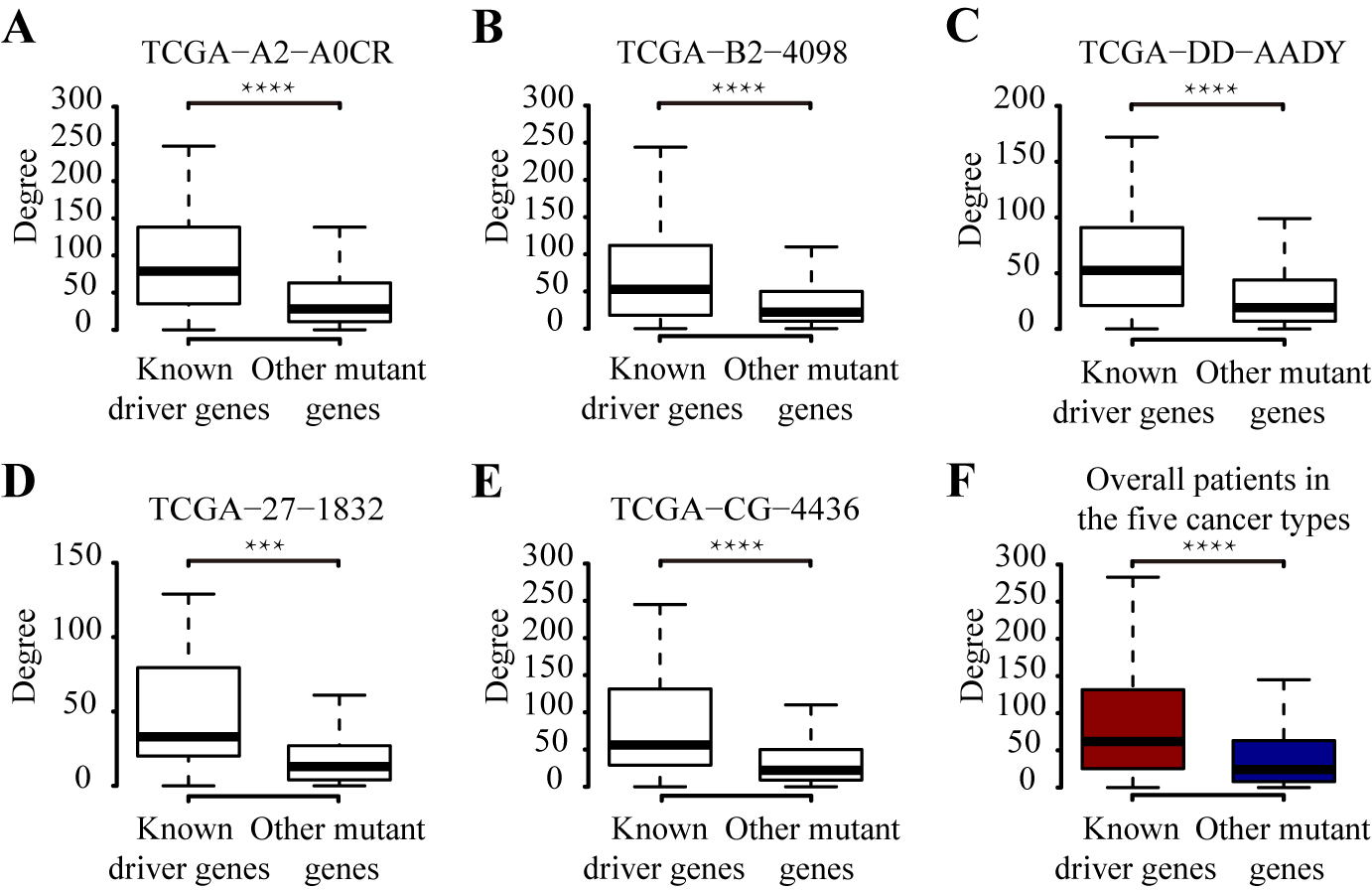

Supplement: S2 Fig — (A-E) Comparison in randomly selected tumor patients. Each subnetwork is induced from STRINGv10 PPI network by the mutant genes and abnormally expressed genes of that patient. (F) Cumulating the result of all the patients in a large cohort consisting of 2022 tumor samples across five cancer types. * P < 0.05, ** P < 0.01 *** P < 0.001 and **** P < 0.0001using the Satterthwaite approximation t test. (TIF) [file pcbi.1012068.s003.tif]

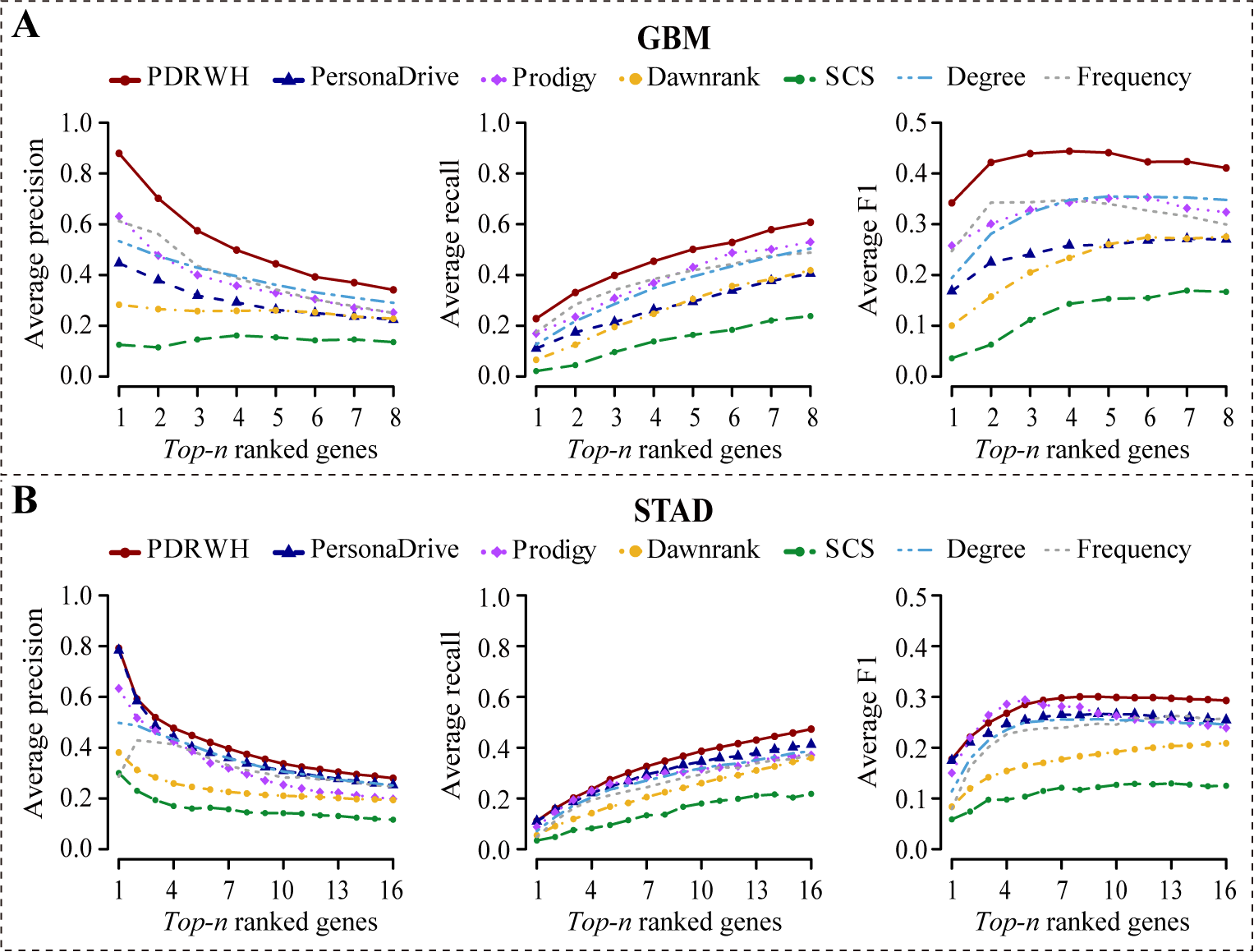

Supplement: S3 Fig — The average precision, recall, and F1-score for (A) the GBM dataset and (B) the STAD dataset, are plotted as a function of the number of top-n ranked genes involved in the calculation of the scores. The general driver gene list is used as the reference set. (TIF) [file pcbi.1012068.s004.tif]

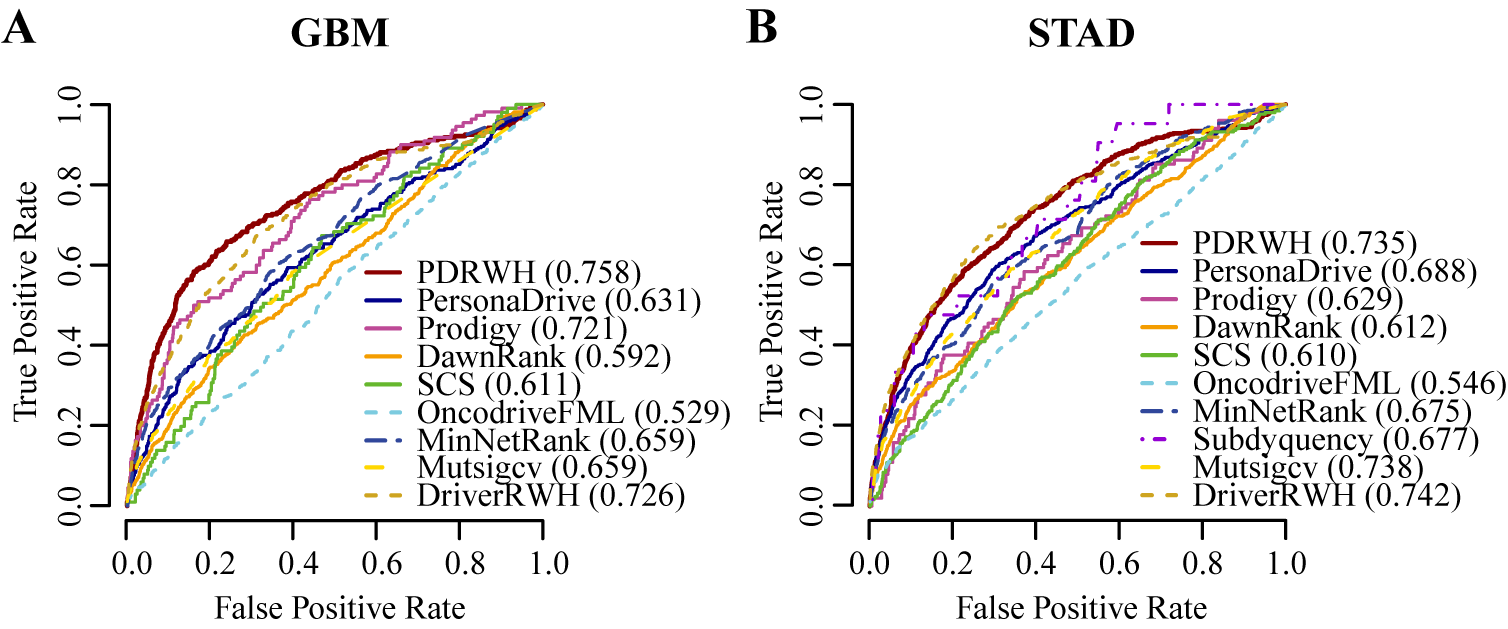

Supplement: S4 Fig — (A-B) ROC plots of results on the five cancer types based on the general reference driver set. The solid lines represent the personalized prediction methods (PDRWH, DawnRank, SCS, PRODIGY and PersonaDrive). The dashed lines indicate the cohort-level prediction methods (OncodriveFML, MinNetRank, Subdyquency, MutsigCV and DriverRWH). The numbers in parentheses behind the methods are corresponding AUC values. (TIF) [file pcbi.1012068.s005.tif]

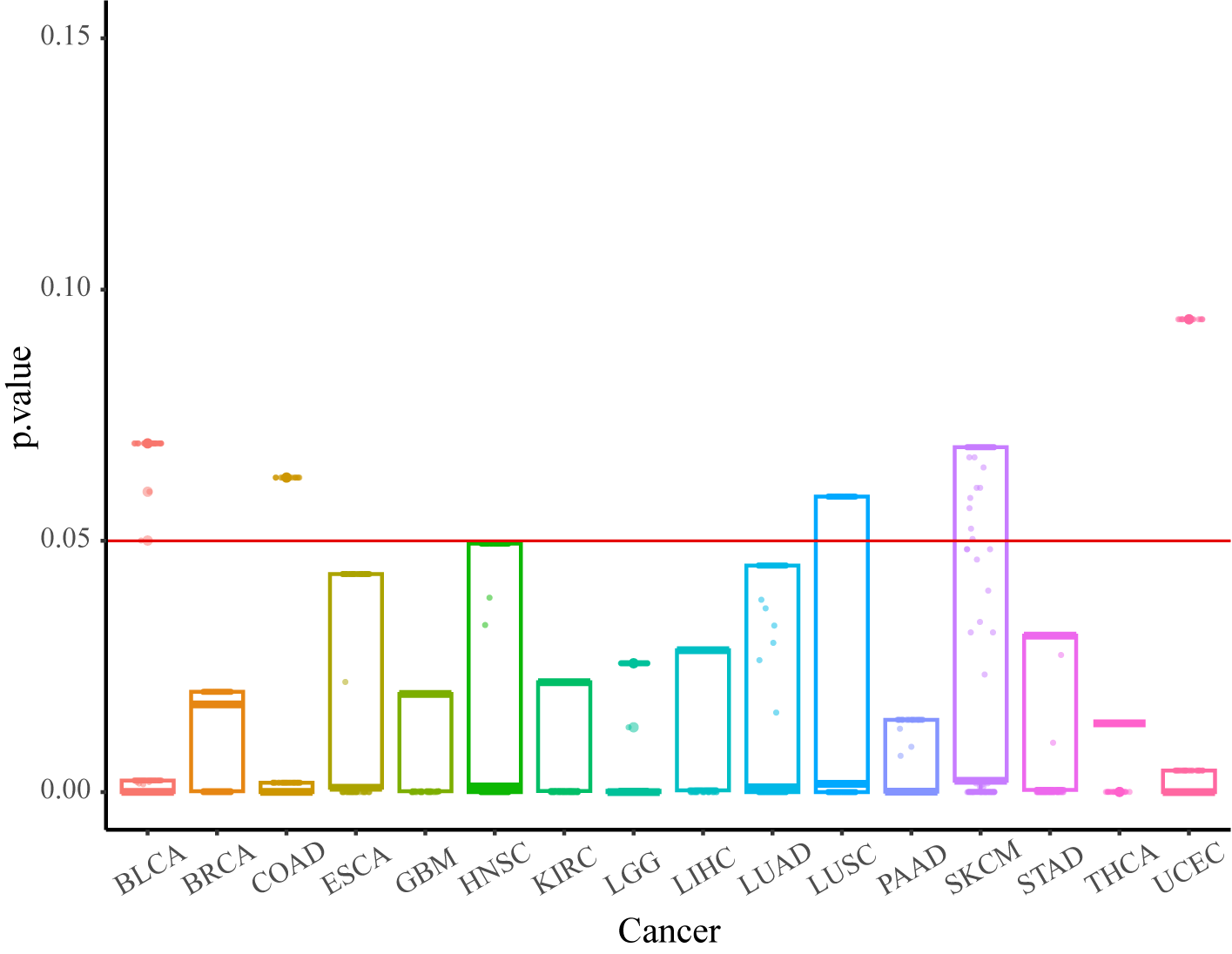

Supplement: S5 Fig — (TIF) [file pcbi.1012068.s006.tif]

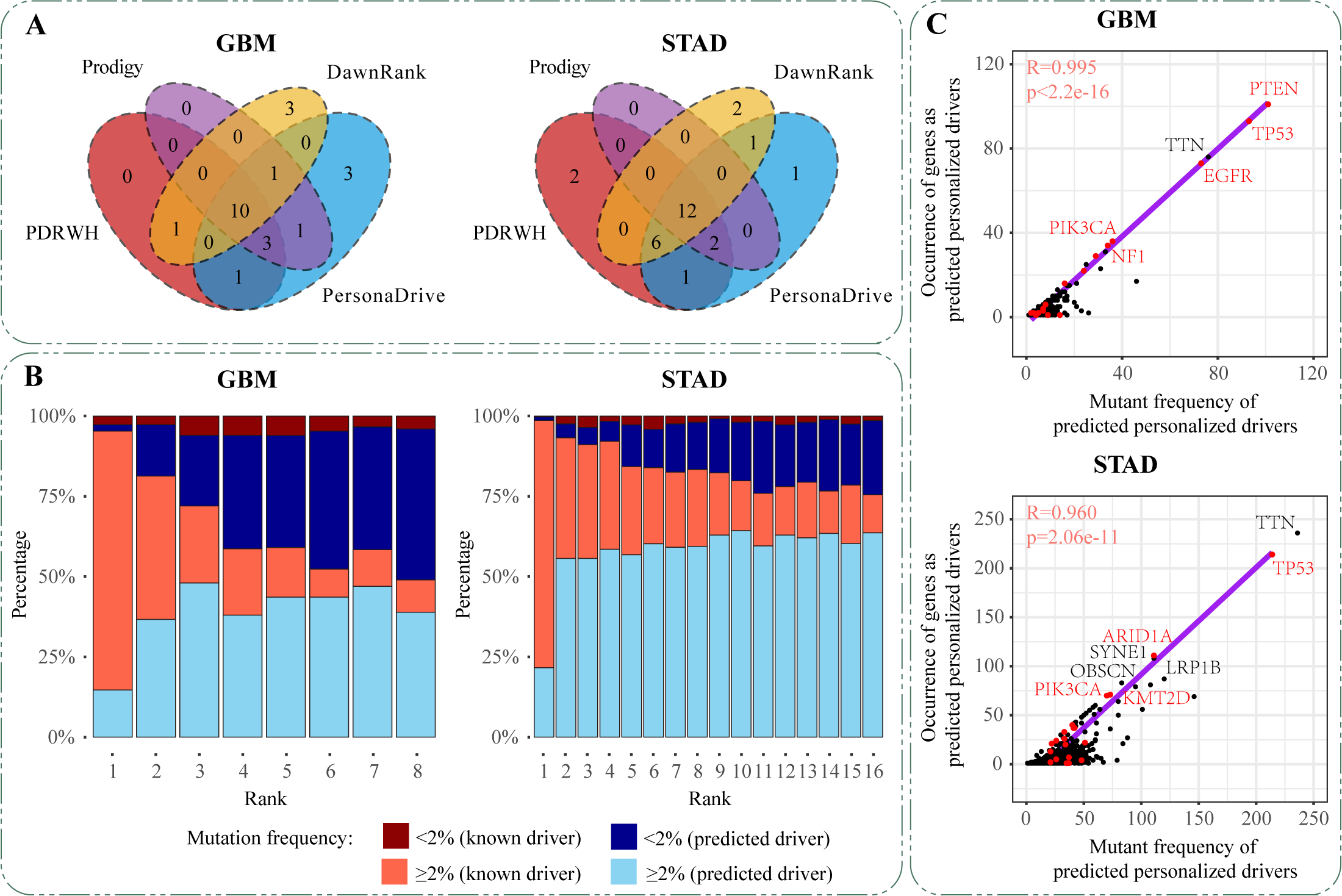

Supplement: S6 Fig — (A) Overlap among the tumor-specific cancer drivers predicted by different methods in GBM and STAD. (B) Distribution of mutation frequency of top genes predicted by PDRWH. The i-th column in the plot represents the distribution of mutation frequency of the genes which ranked at the i-th in the predicted personalized drivers. Each range of mutation frequency is further classified into whether the genes are known drivers in the general reference driver gene list. (C) Scatter plots about mutation frequency of potential drivers and the occurrence of genes as predicted driver gene. Known tumor-specific driver genes are represented as red dots and others are represented as black dots. Purple lines constructed by known tumor-specific driver genes are the regression lines. (TIF) [file pcbi.1012068.s007.tif]

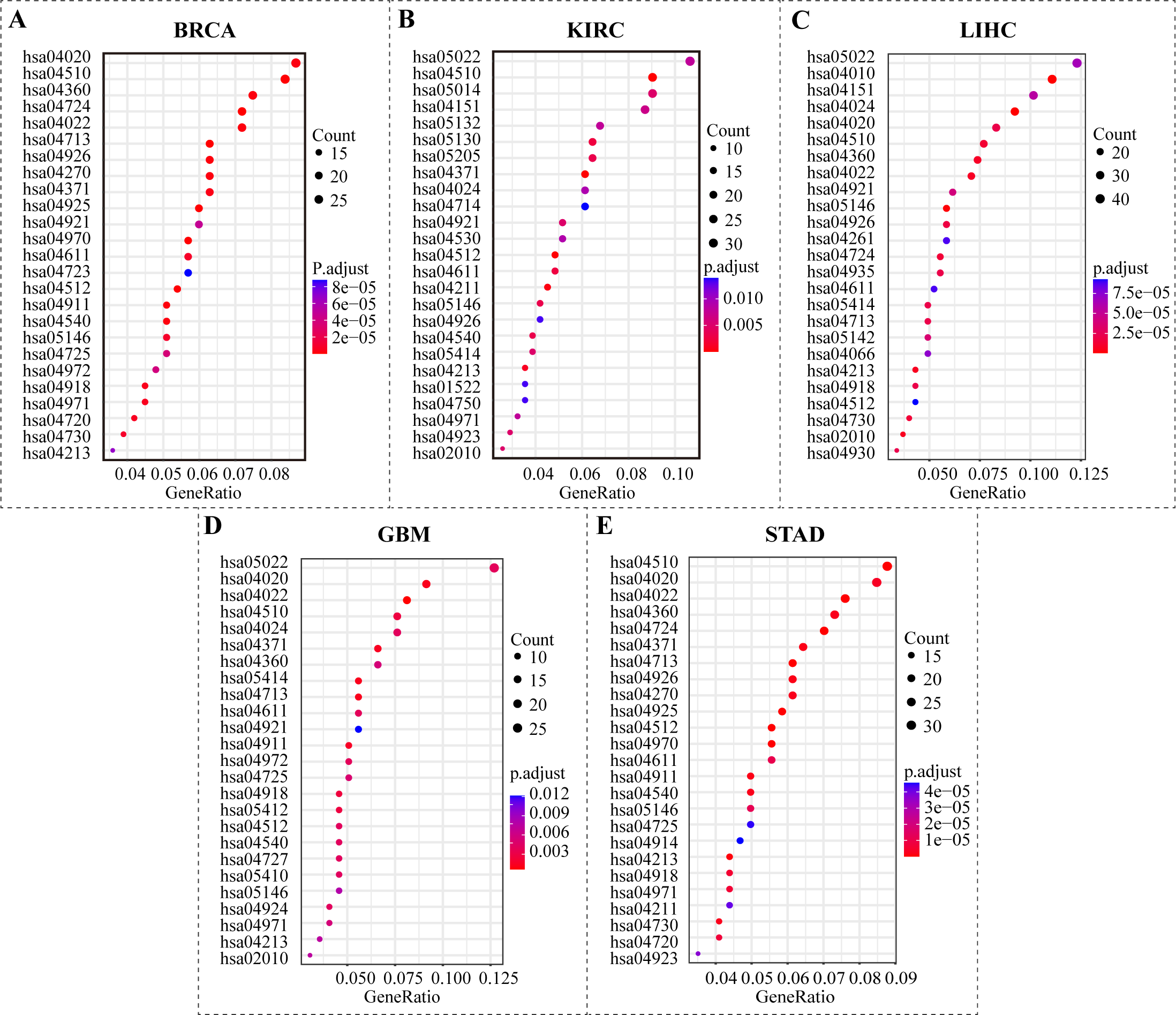

Supplement: S7 Fig — The vertical axis represents the id of KEGG pathway, such as “hsa04020: Calcium signaling pathway” and “hsa05022: Pathways of neurodegeneration-multiple diseases”. The ids and names of KEGG pathways can be found in S4 Table. And “GeneRatio” represents the ratio of the number of genes enriched in the target pathway to the gene list. (A-E) The potential driver genes predicted in the cohort by PDRWH enriched in part of KEGG pathways. (TIF) [file pcbi.1012068.s008.tif]

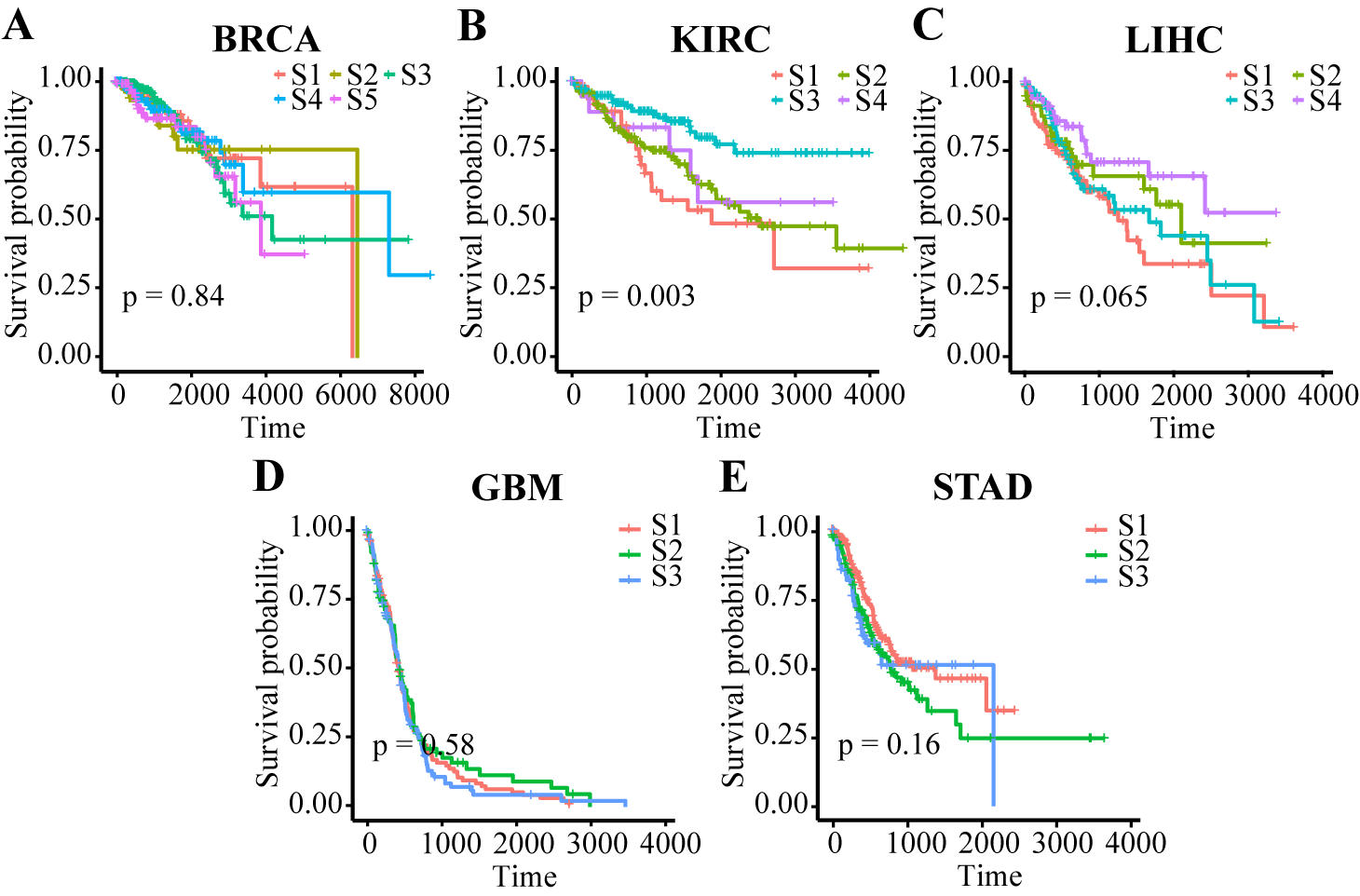

Supplement: S8 Fig — (A-E) Tumor stratification using the gene expression of known tumor specific drivers in BRCA, KIRC, LIHC, GBM and STAD respectively. Different subtypes (S1, S2,…) are indicated by different colored lines. (TIF) [file pcbi.1012068.s009.tif]

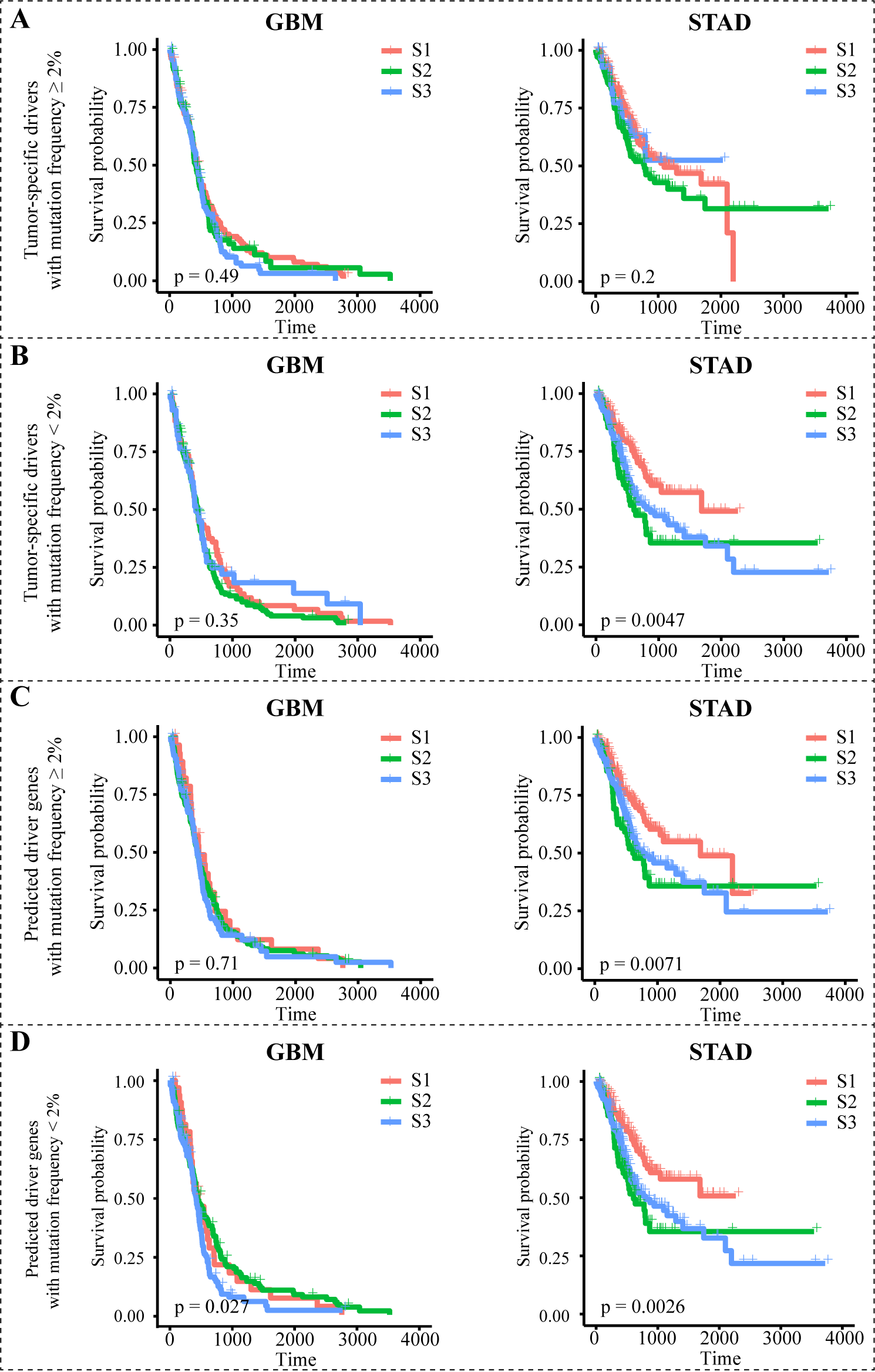

Supplement: S9 Fig — (A) In different cancer types, the expression data of genes those are known tumor-specific drivers with mutation frequency ≥2% were used in subtyping patients. (B-D) Similar analysis based on expression data of genes which are known tumor-specific drivers with mutation frequency <2%, predicted driver genes with mutation frequency ≥2% and predicted driver genes with mutation frequency <2% respectively. Different subtypes (S1, S2,…) are indicated by different colored lines. (TIF) [file pcbi.1012068.s010.tif]

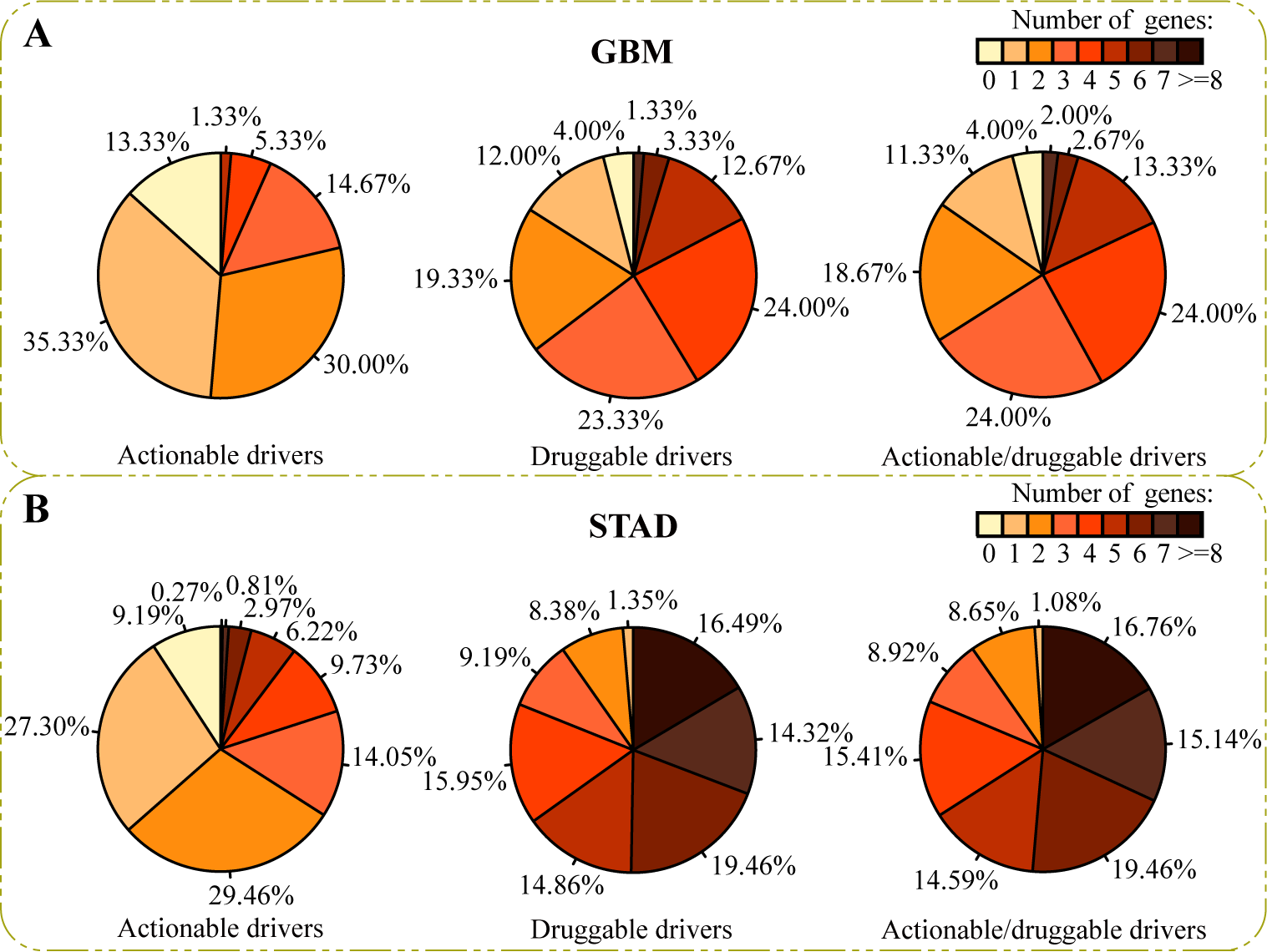

Supplement: S10 Fig — (A) For cancer type GBM, the first pie chart shows the distribution of the number of predicted personalized driver genes in TARGET. Restricted to predicted personalized drivers predicted by PDRWH, there are 21.33% of patients with not less than three actionable driver genes. The second pie chart shows the distribution of the number of predicted personalized driver genes in DGIdb. There are 64.67% of patients with not less than three druggable personalized drivers. The third pie chart is the distribution of the number of predicted personalized driver genes in the union of the two sets. (B) The similar pie chart display for cancer type STAD. (TIF) [file pcbi.1012068.s011.tif]
