# Supplementary material for: A novel hypergraph model for identifying and prioritizing personalized drivers in cancer
Source: PLoS Comput Biol. 2024 Apr 29;20(4):e1012068. doi: 10.1371/journal.pcbi.1012068 (PMC11081510; doi:10.1371/journal.pcbi.1012068)
Supplement: Attachment — Submitted filename: Responses_to_reviewers.docx [file pcbi.1012068.s017.docx]

Dear Editor and Reviewers,

We appreciate the thoughtful comments from you and the reviewers regarding our manuscript titled “A novel hypergraph model for identifying and prioritizing personalized drivers in cancer”. These comments have been extremely valuable and have greatly contributed to the revision and improvement of our manuscript. We have thoroughly reviewed all the comments and have made detailed revisions (modifications in the manuscript are highlighted in red font). We sincerely hope that these revisions meet your expectations and gain your approval. In summary, we have implemented the following major revisions:

1. To highlight the innovativeness of our approach compared to existing methods, we have included a detailed discussion of these methods in the **introduction** section. By analyzing their limitations, we explained the rationale for utilizing the hypergraph model to represent complex high-order relationships among genes.
2. We redesigned **Fig 1** to enhance the clarity of our model. In the revised version, we incorporated a concise example with a small number of vertices to demonstrate the model construction, the weighted assignment mechanism, and the operation process of our algorithm. This example serves as a practical demonstration of how our method works. Additionally, we offered a clearer explanation in the **Overview** section to facilitate a comprehensive understanding of our method.
3. Following the Reviewer’s suggestion, we added more detailed descriptions of the essential conceptual definition and the parameter choices, and an explanation for the evaluation criteria used in our work in the **Methods and Materials** section. In the revised version, we improved the description of the algorithm by dividing it into three main parts and providing detailed explanations for each part. Particularly, we redesigned the section on "Generalized Random Walks" to ensure a smoother and more readable presentation.
4. In the revised version, we have included the performance of our method on 16 cancer datasets from TCGA.  In response to the reviewer's feedback, we re-evaluated the shortcomings of the original evaluation scheme and updated the performance analysis in the Results section using a modified evaluation framework in the **Results** section.

We addressed the individual points raised by the reviewers, as detailed below. The reviewers’ comments are in blue, and our replies are in regular font.

# Review Report 1

## Comments to the Author

The authors proposed a method for identifying and prioritizing personalized cancer driver genes. Their method is capable of discovering high-frequency as well as rare potential drivers. In general, the method is novel and interesting, and the algorithm performs well. However, we request some revisions to strengthen the manuscript before we can support publication.

**Response:**

We appreciate these positive comments by the reviewer.

## Major comments:

1. In the section of the introduction, the authors should clarify why the hypergraph works. What's the biological significance? It will be more complete if the authors provide a brief motivation for using a hypergraph model instead of a graph, such as the limitations of graph models for genes or the intuitive advantages of the hypergraph model.

**Response:**

Thank you for your thorough review. In the revised version, we systematically analyzed the limitations of existing personalized driver gene prediction methods and provided a comprehensive rationale for adopting the hypergraph model. We have highlighted the advantages of the hypergraph model in effectively representing higher-order relationships compared to simple graphs. These additional details serve to enhance readers' comprehension of our design to embrace the hypergraph model and its associated benefits. As suggested, we have incorporated this content into the **introduction** section. We sincerely appreciate your valuable feedback.

(**Introduction** Line 50-82)

“Prodigy prioritizes candidate personalized driver genes by quantifying the impact of mutated genes on deregulated pathway based on the patient’s tumor mutation and expression profiles [19]. According to a single-sample network control strategy, Guo et al. developed SCS to detect the minimum set of driver nodes that could achieve the maximal coverage of individual differentially expressed genes during the transition from the normal state to the disease state [20]. Despite achieving promising results, these personalized driver prioritization methods take into account the data available from a single sample to produce a ranking of drivers for every specific patient, neglecting the availability of data from other samples. More importantly, they are overly dependent on the data quality of individual samples, with poor tolerance for noise and low reliability of the results. To address this issue, PersonaDrive aims to utilize the comprehensive whole cohort data for guiding the personalized driver prediction [21]. This is achieved by constructing a bipartite graph to model pair-wise relationships among the set of mutated genes and the differently expressed genes. However, it is widely acknowledged that the bipartite graph, as a type of simple graph, is limited to capturing pair-wise relationships between nodes and cannot represent more complex relationships. An important aspect of cancer that has been overlooked by existing methods is that patients with the same driver gene mutations are likely to share the same carcinogenic mechanism, unlike patients with only the same passenger mutations [22, 23]. Taking this into account, incorporating higher-order relationships between mutated genes and abnormally expressed genes in computational models holds significant promise for improving personalized cancer driver prediction.

In this study, we present a novel method named PDRWH (Prioritizing Personalized Cancer Driver Genes via Random Walks on a Hypergraph), inspired by the effectiveness of hypergraphs in modeling biology networks, data structures, and other systems [24-27]. Unlike methods that rely on data from a specific sample, PDRWH integrates data from a cohort to generate personalized driver gene predictions, enabling a more comprehensive analysis of the collective information across multiple samples. Under the assumption that the impact of a potential driver gene can be determined by its effect on the genes regulated by it, PDRWH ranks potential driver genes based on the influence of mutated genes on transcriptional networks across the cohort of samples. To achieve this, a hypergraph model is proposed to effectively represent high-order relationships among genes. It captures the implicit intrinsic regulatory associations among genes within each sample by connecting a large number of mutated genes and aberrantly expressed genes in the corresponding hyperedge. Additionally, this model accurately characterizes the coexistence of mutated genes and aberrantly expressed genes across diverse samples. PDRWH quantifies the impact of each mutated gene across the group of samples by performing a generalized random walks algorithm on the personalized hypergraph.”

2. Meanwhile, the author should at least provide some references for the hypergraph since its definition is less intuitive than the normal graphs.

For example, the author could include the following references:

[1] Bretto, A. (2013). Hypergraph theory. An introduction. Mathematical Engineering. Cham: Springer.

[2] Zhang, S., Ding, Z., & Cui, S. (2019). Introducing hypergraph signal processing: Theoretical foundation and practical applications. IEEE Internet of Things Journal, 7(1), 639-660.

[3] Barbarossa, S., & Tsitsvero, M. (2016, March). An introduction to hypergraph signal processing. In 2016 IEEE International Conference on Acoustics, Speech and Signal Processing (ICASSP) (pp. 6425-6429). IEEE.

**Response:**

Thank you for your introduction to these wonderful research works. According to your suggestions, we added these related references in the manuscript as **Reference [24-27]**.

(**Introduction**, Line 69-71)

“In this study, we present a novel method named PDRWH (Prioritizing Personalized Cancer Driver Genes via Random Walks on a Hypergraph), inspired by the effectiveness of hypergraphs in modeling biology networks, data structures, and other systems [24-27].”

3. In the section “Pre-processing”, a gene whose absolute z-score is more than 2 can be selected as an abnormally expressed gene in the sample. This seems to imply that outlying genes are extracted from the population based on their expression values. Are different thresholds considered in this step?

**Response:**

Thanks for your question. We have added details about data normalization and outlier gene screening. In the revised version, we modified the original threshold settings, and have added a description of the z-value threshold selection for screening for abnormally expressed genes in the "**Pre-processing**" section to provide more methodological details.

**(Methods and Materials, Pre-processing**, Line 406-420)

“In a given cohort gene expression profile $X=\{x_{i,j}\}$, where the rows represent genes and columns represent patient samples, the gene expression values are processed using z-score normalization by the following formula:

$x_{i,j}^{'}=\frac{x_{i,j}-\mu_{i}}{\sigma_{i}}$	(1)

where $\mu_{i}$ and $\sigma_{i}$ are the mean expression values and standard deviation of the gene $i$. To identify the abnormally expressed genes for each patient, a threshold $\theta$ is set for each gene, which is the smaller value between the absolute values of the 5% and 95% quantiles among {$x_{i,j}^{'}$}, to pick the significant high or low expression values of the standard gene expression profile $X^{'}$. A gene $i$ is regarded as an abnormally expressed gene of sample $j$ if its absolute value of $x_{i,j}^{'}$ is more than $\theta$, which indicates that the gene is expressed quite differently in this sample compared to the other samples. The advantage of this definition of abnormally expressed genes is that it does not rely on the existence of paired normal and tumor data of the same patient or background gene expression profile from healthy samples. It provides a robust method to detect genes with distinct expression patterns within the cohort. For each sample, the number of genes contained in the set of abnormally expressed genes is approximately about 400 to 1600.”

4. Why does it work better than other algorithms, and what steps work?

**Response:**

Thank you for your question. In the revised **Introduction**, we have provided a detailed analysis of existing personalized driver gene prediction methods, emphasizing their reliance on data from individual samples only, which not only results in wasted data but also fails to achieve the desired predictive performance. Drawing inspiration from PersonaDrive, we explore the use of cohort data to enhance personalized gene prediction. What distinguishes our approach from PersonaDrive is the utilization of a hypergraph model to capture higher-order relationships between genes, going beyond traditional pairwise binary relationships. Due to our model's ability to capture complex relationships between genes, our approach involves using a set of samples that are similar to the target sample, which has contributed to improving the results of personalized driver gene prediction. We have added a detailed description of that in the **Discussion** section.

(**Discussion** Line 362-370)

“The novelty of this study lies in the introduction of the concept and methodology of hypergraph random walks to predict personalized driver genes. The hypergraph model offers substantial benefits in terms of data integration and interpretability. By grouping patient-specific mutated genes and abnormally expressed genes within the corresponding hyperedge, the hypergraph model allows for a comprehensive representation of association among genes across multiple samples simultaneously, rather than separately. The random walk algorithm on the hypergraph is tailored to generate a quantitative assessment of the influence on the gene interaction network within the target sample and its neighboring samples, facilitating the systematic prioritization of candidate personalized driver genes.”

5. The authors should clarify whether a directed or undirected PPI network is used.

**Response:**

We sincerely apologize for any inconvenience caused by the lack of clarity in our previous statement. In the PDRWH method, it is important to note that the protein-protein interaction (PPI) network used is undirected. We have added a description of the network in the revised version. Thank you for bringing this to our attention.

(**Methods and Materials, Construction of personalized hypergraph model,** Line 450)

“PDRWH views the gene network as an undirected graph.”

## Minor comments:

1) In Fig 4b, “%” should be added after the values in parentheses. For example, “33 (84.62%)”.

2) In line 262, the “Supplementary File 2” should be replaced with “S2 File”, to be consistent with the context.

3) The language needs to be improved as some grammatical and spelling errors exist, e.g.,

a) Paper 17 line 259, “is applied to” -> “was applied to”

b) Paper 21 line 290, “and negatively associates” -> “and negatively associate”

c) Page 23 line 324, “to discover” -> “for discovering”

d) Page 29 line 445 “The degree of known driver” -> “The degree of the known driver”

e) Page 31 line 481 “the expression data of genes those are known tumor specific drivers…” is not a complete sentence; “tumor specific” -> “tumor-specific”

**Response:**

Thank you for your thorough review of our paper. We have made the necessary revisions based on your feedback. Additionally, we have checked and corrected other grammar issues in the article. Your valuable comments are greatly appreciated and crucial to our work.

# Review Report 2

## Comments to the Author:

The authors propose a computational method named PDRWH to identify personalized cancer drivers. This is an important problem in cancer genetics as accurate identification of personalized drivers is critical in determining patient-specific therapies. PDRWH ranks mutated genes based on their impact on differentially expressed genes. It also takes into account other patients when building the personalized driver gene list of a patient. PDRWH has been shown to perform better than personalized driver identification methods as well as cohort-level driver identification methods. The authors have also performed additional evaluation steps where they show that PDRWH can identify rare drivers and gene expression of these rare drivers can stratify the patients into different survival groups. Lastly, they provide experimental results that provide support for driver potential of one of the predicted genes, LRP1.

**Response:**

We sincerely appreciate the reviewer for carefully reviewing our paper and offering the positive comments mentioned above.

1. I find the novelty of PDRWH questionable due to the following reasons:

Ranking genes based on their impact on abnormal gene expression is common in the related literature . It has been used in several driver identification methods starting from DriverNet and followed by others e.g. Prodigy, PersonaDrive etc. PersonaDrive is a personalized driver identification method that uses not only the patient of interest but also other patients, so using other patients’ info is also not specific to PDRWH. Many of the driver identification methods that are being compared in this manuscript use random walk strategy. To summarize, PDRWH’s novelty mainly lies in how this random walk is performed in a hyper graph formed by the mutated and “abnormally” expressed genes of each patient. The authors should clarify the contributions of PDRWH more accurately in the Introduction section.

**Response:**

Thank you for your insights on the novelty of PDRWH. Indeed, as the reviewer pointed out, ranking genes based on their impact on abnormal gene expression is a common practice in driver identification methods. It has been used in several driver identification methods starting from DriverNet and followed by others like Prodigy and PersonaDrive. Although PersonaDrive is also an example of utilizing information from other patients for predictions, it employs a simple graph model that cannot capture the complex relationships among genes. The advantage of the hypergraph model used in our method lies in its ability to effectively integrate the association among genes within individual samples and across samples into a unified framework. resulting in improved accuracy and comprehensiveness of gene driver identification by PDRWH. This enhancement enables PDRWH to elevate the accuracy and comprehensiveness of gene driver identification. In our revised version, we systematically analyzed the limitations of existing personalized driver gene prediction methods and provided a comprehensive rationale for adopting the hypergraph model. We have highlighted the advantages of the hypergraph model in effectively representing higher-order relationships compared to simple graphs. These additional details serve to enhance readers' comprehension of our design to embrace the hypergraph model and its associated benefits. As suggested, we have incorporated this content into the **introduction** section. We sincerely appreciate your valuable feedback.

(**Introduction** Line 50-82)

“Prodigy prioritizes candidate personalized driver genes by quantifying the impact of mutated genes on deregulated pathway based on the patient’s tumor mutation and expression profiles [19]. According to a single-sample network control strategy, Guo et al. developed SCS to detect the minimum set of driver nodes that could achieve the maximal coverage of individual differentially expressed genes during the transition from the normal state to the disease state [20]. Despite achieving promising results, these personalized driver prioritization methods take into account the data available from a single sample to produce a ranking of drivers for every specific patient, neglecting the availability of data from other samples. More importantly, they are overly dependent on the data quality of individual samples, with poor tolerance for noise and low reliability of the results. To address this issue, PersonaDrive aims to utilize the comprehensive whole cohort data for guiding the personalized driver prediction [21]. This is achieved by constructing a bipartite graph to model pair-wise relationships among the set of mutated genes and the differently expressed genes. However, it is widely acknowledged that the bipartite graph, as a type of simple graph, is limited to capturing pair-wise relationships between nodes and cannot represent more complex relationships. An important aspect of cancer that has been overlooked by existing methods is that patients with the same driver gene mutations are likely to share the same carcinogenic mechanism, unlike patients with only the same passenger mutations [22, 23]. Taking this into account, incorporating higher-order relationships between mutated genes and abnormally expressed genes in computational models holds significant promise for improving personalized cancer driver prediction.

In this study, we present a novel method named PDRWH (Prioritizing Personalized Cancer Driver Genes via Random Walks on a Hypergraph), inspired by the effectiveness of hypergraphs in modeling biology networks, data structures, and other systems [24-27]. Unlike methods that rely on data from a specific sample, PDRWH integrates data from a cohort to generate personalized driver gene predictions, enabling a more comprehensive analysis of the collective information across multiple samples. Under the assumption that the impact of a potential driver gene can be determined by its effect on the genes regulated by it, PDRWH ranks potential driver genes based on the influence of mutated genes on transcriptional networks across the cohort of samples. To achieve this, a hypergraph model is proposed to effectively represent high-order relationships among genes. It captures the implicit intrinsic regulatory associations among genes within each sample by connecting a large number of mutated genes and aberrantly expressed genes in the corresponding hyperedge. Additionally, this model accurately characterizes the coexistence of mutated genes and aberrantly expressed genes across diverse samples. PDRWH quantifies the impact of each mutated gene across the group of samples by performing a generalized random walks algorithm on the personalized hypergraph.”

## Other major issues:

1. The authors use Prodigy’s evaluation scheme in Fig 2. PersonaDrive paper lists some disadvantages of this evaluation scheme and propose a modified version. It would be useful to see PDRWH’s performance with this modified evaluation scheme.

**Response:**

Thank you for pointing out the potential value in evaluating PDRWH's performance with the modified evaluation scheme proposed in PersonaDrive. This comparison could have offered valuable insights into the effectiveness of PDRWH in a more refined evaluation framework. We have replaced the comparison scheme in the “**Methods and Materials”** section with the modified version as advised by you and have also updated the results in **Fig 2**. Thank you for your valuable suggestion.

(**Methods and Materials, Comparison to other methods,** Line 511-513)

“We used the modified REA strategy proposed by PersonaDrive [21] for a comparison of PDRWH with six personalized prediction methods (DawnRank, Prodigy, SCS, PersonaDrive, Degree and Frequency) across five cancer types from TCGA.”

2. I think the evaluations should include two baseline driver identification methods that ranks the mutated genes of a patient:

a) based on their degree in the interaction network;

b) based on the number of patients that this gene is mutated in.

**Response:**

Thank you for your suggestion. In **Fig 2**, we have added two additional baselines—one based on network degree and the other on mutation frequency. Our observation revealed an interesting phenomenon: Methods that utilize cohort sample information (PDRWH, PersonaDrive, and Frequency) demonstrate significantly better performance compared to those using single sample data (Prodigy, DawnRank, and SCS). Notably, our method, which integrates both frequency and degree information, outperforms all other approaches. It is evident that including these two baseline methods effectively demonstrates the impact of using different data information on algorithm results, making it a very commendable idea. In the original text, we added the following explanation at the corresponding position:

(**Results, PDRWH outperforms existing driver gene prediction methods in identifying known general drivers,** Line 171-183)

“At the same time, to highlight the impact of utilizing different data information on algorithm results, two naive methods were introduced as baselines. One method ranks genes based on the mutation frequency among samples, while the other method ranks genes based on the degree in the gene subnetwork induced by mutated genes within the sample. As mentioned above, we utilized the general driver gene reference as the benchmark of known drivers. From **Fig 2** and **S3 Fig**, we found that methods utilizing the cohort data (PDRWH, PersonaDrive, and Frequency) consistently outperformed those relying on individual data (Prodigy, DawnRank, and Degree) in terms of average precision, recall, and F1-score. This highlights the significant advantage of leveraging collective data over individual data. Among the three cohort data analysis methods (PDRWH, PersonaDrive, and Frequency), PDRWH exhibits superior performance, achieving an outstanding precision rate of up to 88.4% and 76.0% for genes ranked first in BRCA and KIRC. Although PDRWH's precision for the top-ranked gene in KIRC is slightly lower than PersonaDrive, its superiority becomes more noticeable as the ranking advances, particularly in terms of recall and F1-score.”

(**Fig 2**)

**Fig 2. Comparison of the PDRWH with the other personalized prediction methods.**

The average precision, recall, and F1-score for (**A**) the BRCA dataset, (**B**) the KIRC dataset, and (**C**) the LIHC dataset, are plotted as a function of the number of *top-n* ranked genes involved in the calculation of the scores. The general driver gene list is used as the reference set.

(**S3 Fig**)

**S3 Fig. Comparison of the PDRWH with other personalized prediction methods.**

The average precision, recall, and F1-score for (**A**) the BRCA dataset, (**B**) the KIRC dataset, and (**C**) the LIHC dataset, are plotted as a function of the number of *top-n* ranked genes involved in the calculation of the scores. The general driver gene list is used as the reference set.

3. PDRWH determines neighbors of a patient via the concept of shared mutated genes. The authors should include a table with statistics on the number of neighbors for each patient as I would expect a very dense graph using this definition.

**Response:**

Thank you for your suggestion. We have incorporated a histogram **S1 Fig**, illustrating the distribution of neighbors for each sample across different cancer types. We found that the vast majority of patients have more than 50 neighbors, indicating that our approach can generate a relatively dense personalized hypergraph for most patients. Adding this figure can give readers an intuitive impression of how many samples are involved in the prediction of personalized driver genes for each sample. I sincerely appreciate the insightful suggestion from the reviewer once again.

(**Results, An overview of PDRWH**, Line 108-109)

“As depicted in S1 Fig, each hypergraph typically consists of more than 50 hyperedges (samples), indicating that PDRWH generates a relatively dense personalized hypergraph for most individuals.”

(**S1 Fig**)

**S1 Fig. The distribution of neighbor numbers involved in modeling a personalized hypergraph.** The n in parentheses represents the number of tumor samples.

## Minor issues:

1) It would be helpful to provide a toy hyper graph in “Construction of personalized hypergraph” to better explain how weights are assigned to vertices and edges.

**Response:**

Thank you for your suggestion. We have redesigned **Fig 1** to include a toy hypergraph with a small number of vertices and hyperedges. This helps clarify some details of our algorithm, such as how to assign weights to vertices and hyperedges. Additionally, we have revised the overview section in the original text. We believe that this will provide readers with a better understanding of our approach, and we thank the reviewers for their helpful feedback.

(**Results, An overview of PDRWH,** Line 91-123)

“**An overview of PDRWH**

The PDRWH method is a novel integrated genome/transcriptome analysis approach designed to identify candidate personalized driver genes by leveraging the influence of mutated genes on biological networks across cohort samples. We hypothesize that samples with the same cancer types of cancer display higher similarity in molecular characteristics and disease mechanisms compared to samples with different types of cancer. Leveraging samples from the same cancer type, specifically those closely resembling the target sample, has the potential to improve the personalized driver genes prediction. A schematic overview of PDRWH is illustrated in **Fig 1**. PDRWH requires knowledge of cohort samples from a specific cancer type in TCGA, including the somatic mutation and the gene expression profiles, as well as a gene interaction network (**Fig 1A**). To ensure uniform baseline values and ranges for all genes across the cohort samples, gene expression data is normalized. Subsequently, a screening process is conducted to identify abnormally expressed genes for each sample (**Fig 1B**). PDRWH consists of three main steps. The first step involves constructing a personalized hypergraph model, where samples are represented as hyperedges, and the mutated genes as well as the abnormally expressed genes in the target sample are described as vertices (**Fig 1C**). In this model, a hyperedge in the hypergraph is capable of connecting multiple vertices (not limited to two nodes as in a simple graph), enabling a more complex representation of relationships between genes and facilitating a comprehensive analysis of association across the cohort samples. As depicted in **S1 Fig,** each hypergraph typically consists of more than 50 hyperedges (samples), indicating that PDRWH generates a relatively dense personalized hypergraph for most individuals. Following this, weights are assigned to hyperedges based on the Pearson correlation of gene expression profiles between the corresponding samples and the target sample. This weighting scheme ensures that patients who closely resemble the target patient have a more substantial impact on the personalized driver gene prediction. The weights of vertices within each hyperedge are set as the degree of the subnetwork induced by genes corresponding to these vertices, characterizing the influence of mutated genes on the abnormally expressed genes in each sample. In the second step, the transition probability matrix is calculated by taking into account the structure of the hypergraph as well as the weights of the hyperedges and vertices. This matrix indicates the probabilities of transitioning from one vertex to another within the hypergraph (**Fig 1D**). The final step entails performing a restart random walk on the weighted personalized hypergraph. After the random walks reach a stable state, a stationary distribution of the random walk is achieved. At this point, the PDRWH-scores are generated to quantitatively and rationally prioritize candidate genes (**Fig 1E**). For a target patient, a mutated gene in the target sample should be ranked higher if it is adjacent to many genes that are abnormally expressed in the target sample as well as in a group of other samples from the same cancer type.”

(**Fig 1**)

**Fig 1. Overview of PDRWH for prioritizing personalized cancer driver genes.**

(**A**) Model input. i) The somatic mutation profiles from the TCGA; ii) The gene expression data of patients; iii) Gene interaction network. (**B**) Pro-processing the gene expression profiles and determining the abnormally expressed genes for each sample. (**C**) Construction of a hypergraph model for each patient. In this model, each hyperedge represents a patient and the vertices incident to each hyperedge represent the mutated genes and abnormally expressed genes in the corresponding patient. The weights of the hyperedges are determined based on the gene expression similarity between samples. Meanwhile, the weights of vertices in each hyperedge are assigned according to their connection in the corresponding gene interaction subnetwork. (**D**) Computing the transition probability matrix of the random walks on the weighted hypergraph. (**E**) The process of generating PDRWH-scores through random walks on the personalized hypergraph. The walker, starting at a present vertex 𝑢, firstly chooses a hyperedge with probability determined by the weights of hyperedges, and then travels towards any vertex (for example *v*) within the selected hyperedge based on the weights of the vertex in the hyperedge.

2) The authors should explain how this is achieved “In this case, the walker will be more likely to stabilize at the vertex showing a higher degree of interaction not only in the subnetwork of the target sample but also in those of its neighbors”

**Response:**

Thank you for your advice. In the revised **Fig 1**, the walker’s preference for vertices showing a high degree of interaction not only in the subnetwork of the target sample but also those of its neighbors can be explained as follows:

When the walker traverses the hypergraph, it considers both the subnetwork of the target sample and the subnetworks of its neighboring samples. For the target sample, we designed two vertices in the corresponding PPI subnetwork, node 2 and node 5. However, since node 2 also has a relatively high degree in the subnetworks of neighboring samples, it receives a higher preference compared to node 5.

By adding such an example, readers can intuitively appreciate the rationale behind the algorithm design. We greatly appreciate any suggestions provided by the reviewers to include a toy model (in **Fig 1C-E**), and we once again express our gratitude for this suggestion.

3) I don’t understand why “potential” is included in the section “PDRWH efficiently identifies both potential frequent and rare drivers.

**Response:**

Thank you for your feedback. We have carefully reviewed and revised the text to ensure the inappropriate use of the word "potential" has been removed. Additionally, we have revised the title of this section and provided detailed explanations of the definitions of the common and rare genes. Your guidance has been truly valuable and appreciated.

(**Results, PDRWH efficiently identifies both common and rare drivers,** Line 250-255)

“**PDRWH efficiently identifies both common and rare drivers**

One of the advantages of our method is its ability to identify both common and rare driver genes, demonstrating a balanced performance that effectively trades off algorithm generalizability and specificity. To further demonstrate this ability, we divided the top-ranked predicted driver genes into two categories based on the frequency of occurrence in the respective cancer-type cohort: common and rare. Genes with mutation frequency $\geq$2% are classified as common drivers, while those with a mutation frequency <2% are classified as the rare. The results are summarized in **Figs 5A-C and S6B Fig.**”

4) It’s not clear how k is determined in k-means algorithm for tumor stratification analysis.

**Response:**

In the revised version, an explanation of how we determined the number of categories (k) in our research has been included.

(**Results, Subtypes recovered by expressions of predicted rare drivers are significantly associated with patient survival,** Line 284-286)

“Employing unsupervised K-means clustering, we separated the tumor samples into different subtypes based on the gene expression of the predicted personalized drivers. The number of clusters for each cancer type was determined using a CDF (Cumulative Distribution Function) curve.”

5) I couldn’t find for which cancer types LRP1 is identified as a driver. Also, whether this gene is found only by PDRWH should be clarified.

**Response:**

In the revised manuscript, we have corrected the statement. In gastric cancer (GC), the gene LRP1 was predicted as a personalized driver gene for several patients. In fact, through the analysis of PDRWH and PersonaDrive, LRP1 was ranked in the top ten in the target patients, while other methods failed to identify it in GC. Although it is not a known driver gene according to the literature, we conducted some subsequent experimental verification to explore the impact of LRP1 on the development of gastric cancer.

(**Results, Experimental validation of predicted novel cancer drivers,** Line 330-333)

“By way of illustration, Low-density lipoprotein receptor-related protein 1 (LRP1) was predicted as a driver gene in Human gastric cancer cells (GC) by our method and PersonaDrive. This gene was not detected by widely used methods, such as DawnRank, Prodigy, and SCS, and was not presented in the known driver gene list.”

5. English of the manuscript is problematic and has to be improved significantly. Listing only a few:

-…sample tend to adjacent more…

-… Since all the mutant genes in the target patient should be supposed...

-Therefore, the weight of hyperedge should be an increased function with their correlation to target patient.

-we got no satisfactory results

-on the decline at the mRNA and protein levels with transfection of three siRNAs

**Response:**

We appreciate your thorough review of our paper and the valuable reminders you provided. We have implemented the necessary corrections based on your feedback. Additionally, we have improved and enhanced the overall English writing in the revised version. Thank you for your guidance and assistance in refining our work.

# Review Report 3

This paper introduces a computational method tailored for the personalized identification of cancer driver genes, with its foundation grounded in two critical observations:

- Driver alterations typically induce substantial transcriptional changes in genes located upstream or downstream of signaling pathways. In this context, both the driver gene and the associated upstream and downstream genes closely interact with one another.

- Patients sharing comparable tumor transcriptional profiles exhibit analogous tumor progression, suggesting the implication of shared driver genes in their respective cases.

While the overall concept is innovative and founded on robust biological observations, the paper's clarity is compromised. The lack of essential conceptual definitions, rationale behind specific input choices, and explanations for chosen validation methods contribute to the difficulty in comprehension. Additionally, numerous instances of ambiguous grammar further hinder understanding. Addressing these issues would significantly enhance the paper's accessibility and impact. See comments below:

**Response:**

We appreciate these comments from the reviewer. The statement about the conceptual definitions, the rationale behind specific input choices, and explanations for chosen validation methods were revised according to your suggestions. Thank you again for your nice advice.

1) How were mutated genes defined?

a. Was it based on any type of mutation or just non-synonymous mutations?

b. Were focal copy number alterations also considered?

c. What was the rationale behind these decisions?

**Response:**

Thanks for your question. Our method primarily focuses on non-synonymous point mutations and insertions/deletions (indels) in coding regions. These types of mutation are widely utilized in driver gene prediction methods due to their high occurrence rates, ease of detection, and potential as genetic markers for specific phenotypes associated with tumor formation. We have added the following descriptions of the mutation data used in our method.

(**Methods and Materials**, **Data resources**, Line 393-395)

“In this work, we use two types of genomic data from a cohort: somatic mutation data, which includes non-synonymous point mutations and insertions/deletions (indels) in coding regions, and gene expression profiles.”

(**Discussion,** Line 381-386)

“Additionally, our current model primarily focuses on point mutations, including single-nucleotide variants (SNVs) and short insertions or deletions (indels), due to their prevalence, ease of detection, and potential as genetic markers for specific phenotypes that promote tumor formation. However, the impact of other somatic alterations like amplifications, genomic rearrangements, and epigenetic silencing, which are also crucial in tumorigenesis, has not been considered. Integrating information on all these alterations would improve the identification of driver genes in cancer.”

2) To facilitate personalized identification of cancer drivers, the authors propose a hypergraph model, wherein each patient represents a hyperedge connecting two or more mutated/upregulated/downregulated genes in that patient. Each hyperedge in the hypergraph model gets assigned a weight, which is a function of the correlation between the expression profile of a target patient and expression profiles of its neighbors.

a. A clear definition of how the correlation coefficient was computed is missing.

b. A clear definition of the neighbors of a target patient is missing.

c. Basic intuition suggests that the weights assigned to each hyper edge in the hypergraph should be unique for each target patient. However, the equations defined in the paper seem to suggest a fixed set weights for all hyper edges. This is very counter intuitive considering that the authors claim to predict personalized drivers.

**Response:**

I apologize for the lack of clarity in the original text regarding the points mentioned above. In the revised version, we have included detailed explanations of the correlation coefficient and the neighbors of a target patient. Based on the definition provided above, it is evident that the weight of a hyperedge for a target sample is contingent upon the similarity of gene expression between the target sample and its neighbors, as determined by the Pearson correlation coefficient. Consequently, the selection of neighboring samples and their respective weights varies for different target samples.

(**Methods and Materials**, **construction of personalized hypergraph model,** Line 430-447)

“The incidence matrix $H\in\mathcal{R}^{|V|\times|E|}$ is defined as follows:

$h\left( v,e \right)=\left\{ \begin{aligned} 1, if v\in e \\ 0, otherwise \end{aligned} \right.$	(2)

which indicates whether vertices are incident with the hyperedges. According to the assumption that patients with similar gene expression profiles may have the more similar pathogenic mechanism to each other than the rest of the patients, the most closely related patient in terms of its gene expression profile will contribute much more to the prediction of driver genes compared to other patients. Therefore, the weight of a hyperedge should be an increased function with their correlation to the target sample. A fairly standard choice for the weights is:

$w_{e}=exp\left( -\frac{\left\| 1-\rho(e, e_{o}) \right\|^{2}}{2\delta^{2}} \right), e\in E$	(3)

in which $\rho(e, e_{o})$ is the Pearson correlation of gene expression profiles between sample $s$ and the target sample $s_{0}$, and $\delta$ is the bandwidth parameter (default $\delta=0.1$) controlling how quickly the weight of a sample falls off with the distance of $s$ from the query point $s_{0}$. Here, if $\rho(e, e_{o})$ is close to 1, $w_{e}$ will also be close to 1, implying that this sample has a high impact in the evaluation of driver genes of target sample $s$. On the contrary, if $\rho(e, e_{o})$ is small (e.g., close to 0), $w_{e}$will be relatively small too. In this case, the corresponding neighbor will have a weak contribution to the determination of the driver genes of the target sample. Then the weight matrix of hyperedge is defined as the diagonal matrix:

$W_{e}=diag\left\{ w_{e} | e\in E \right\}$	(4)

”

(**Methods and Materials**, **construction of personalized hypergraph model,** Line 424-425)

“For a target sample $s_{0}$, if a sample shares at least one co-mutant gene with this one, it is defined as a neighbor of that patient. Let $S=\{s_{1},\ldots,s_{n}\}$denote the set of neighbor samples of the target sample.”

3) The section describing the Generalized Random Walks is written in a highly convoluted and incoherent language that may make it difficult to follow for other readers.

**Response:**

In the revised manuscript, we have refined the description of the Generalized Random Walks section by splitting it into two parts to ensure clarity, coherence, and readability. Thank you for pointing out the lack of continuity in the previous version. Your feedback is valuable in improving the quality of our work.

(**Methods and Materials**, Line 463-498)

“**Transition probability matrix of the random walks on hypergraph**

A random walk on a hypergraph $H\left( V, E \right)$ is similar to the classic random walk, wherein transitions occur between two incident vertices in the hyperedge [45]. Specifically, the movement between vertices is modeled as a discrete-time Markov chain based on predefined transition probabilities. A standard formulation for a hypergraph random walk can be broken down into two steps. Given the current state $\vec{v}_{t}$:

(i) Starting at a vertex $u$, a hyperedge is selected with a probability determined by the weights of hyperedges $w_{e}$.

(ii) a vertex $v$ is chosen from the selected hyperedge $e$. The walker can travel to any nodes within the selected hyperedge based on the weights of the vertex in the hyperedge $w(v,e)$.

Thus, transition probabilities from vertex $u$ to vertex $v$ are calculated as follows:

$p(u,v)=\sum_{e\in E} \frac{h(u,e)w_{e}}{\sum_{\hat{e}\in E} h(u,\hat{e})w_{\hat{e}}}\frac{w(v,e)}{\sum_{\hat{v}\in V} w(\hat{v},e)}$	(8)

which can be written in an alternative matrix form:

$P=D_{v}^{-1}HW_{e}D_{ve}^{-1}{W_{v}}^{T}$	(9)

where $H$, $W_{e}$ ,and $W_{v}$ are defined as previously mentioned, while $D_{v}$ and $D_{ve}$ represent the diagonal matrix for the degrees of vertices and hyperedges, with $d(v)$ and $\delta(e)$ being the respective diagonal elements.

**Generalized random walks on the personalized hypergraph model**

We implement a restart random walk on the personalized hypergraph by adding a damping factor. Specifically, all the mutated genes in the target sample $s_{0}$are assumed to have an equal probability of being driver genes. Therefore, an equal probability of $\frac{1}{n}$ is assigned to each of the mutated genes in $s_{0}$initially, where $n$ represents the number of mutated genes in $s_{0}$. The initial values of the abnormally expressed genes are set to zero. Let the initial column vector be denoted as $\vec{v}_{0}\in\mathcal{R}^{|V|}$. The process can be mathematically represented by the following formula:

$\vec{v}_{t+1}=\alpha P^{T}\vec{v}_{t}+\left( 1-\alpha\right)\vec{v}_{0}, t\in N$	(10)

where the $i$-th element of state $\vec{v}_{t}$ represents the probability that the walker moves to node $i$ at step $t.$ The damping factor, $\alpha(0<\alpha<1)$, is introduced to ensure the graph satisfies ergodic conditions [45]. In our study, we have empirically set the damping factor $\alpha$ to 0.85. The term $\alpha P^{T}\vec{v}_{t}$ in the formula means that the random surfer may transition to one of the adjacent vertices, while $(1-\alpha)\vec{v}_{0}$ represents a vector introducing the probability of teleporting the random walk back to the initial state. After several iterations of the random walk, the distribution vector $\vec{v}$ stabilizes when the difference between $\vec{v}_{t+1}$ and $\vec{v}_{t}$ measured by L1 norm falls below a small $\varepsilon$ (default ${10}^{-6}$). PDRWH algorithm generally converges within ten times iterations. The stationary probability indicates the likelihood of genes being personalized cancer drivers of the target sample. Finally, the values of mutated genes in the stationary probability vector are preserved and then normalized to generate the PDRWH-score. PDRWH-scores can be ranked in descending order to prioritize the personalized candidate driver genes.”

4) For validation, the authors test how well-known cancer type-specific drivers are enriched among the top-ranked driver genes predicted by their method and benchmark against other methods. However, some additional clarifications are needed on how the experiments was performed.

**Response:**

We appreciate your feedback. We have provided additional details on the experimental procedures to offer comprehensive information about the methods and steps involved.

(**Methods and Materials**, **Comparison to other methods,** Line 526-538)

“The second benchmark involves the identification of tumor-specific driver genes. As there is a remarkable discrepancy among different cancer types, we downloaded a list of tumor-specific driver genes from the IntOGen database [47]. This list is considered to be the best trade-off between sensitivity and specificity among those currently available (S3 File). Given the *top-*$n$ predicted personalized drivers, an enrichment analysis of the personalized driver genes was performed using the hypergeometric test:

$P(X=m)=\frac{\binom{M}{m}\binom{N-M}{n-m}}{\binom{N}{n}}$	(14)

where $N$ represents the total number of genes in a patient, $M$ is the number of genes in the known tumor-specific driver gene list, $n$ is the number of predicted personalized driver genes of the patient, and $m$ is the number of overlapping genes between the known tumor-specific driver genes and predicted personalized driver genes of the individual. If $P\left( X\geq m \right)<0.05$, it indicates that the predicted driver genes for this patient is significantly enriched in known driver genes. Additionally, we investigated the consistency and differences in the identified tumor-specific driver genes among different methods.”

a. What was the rationale behind the choice of only 5 cancer types from TCGA: BRCA, KIRC, LIHC, GBM, STAD? What about other cancer types?

**Response:**

We have modified the description of the **Data resources** in the paper to indicate that the algorithm was utilized for 16 cancer datasets in the TCGA. Given the limited space in the article, we have chosen to focus on five common types of cancer as examples to demonstrate the evaluation of the algorithm's performance in the main results. Nonetheless, our approach exhibits notable performance across various other cancer types. We have included the results for 16 different types of cancer in the **S2 Table** and **S5 Fig** to demonstrate its performance across a broad of cancer types.

**(Results, PDRWH outperforms existing driver gene prediction methods in identifying known general drivers,** Line 156-163)

“We evaluated the performance of PDRWH's ability to identify known general driver genes based on the top-ranked genes. As shown in the **S2 Table**, in as many as 85% of the total samples, the genes ranked first by PDRWH are known driver genes. Our approach shows exceptional performance in identifying known driver genes, particularly when considering genes at the top of the predictions, except in SKCM. In LGG and UCEC, this proportion even reaches up to 95.8% and 98.2%. On the whole, approximately 50% of the samples have the gene ranked second as a known driver gene. As the ranking increases, the proportion of samples where these genes are known driver genes decreases, just as we expected.”

(**Methods and Materials**, **Data resources,** Line 396-398)

“We downloaded 16 cancer datasets that contained a sufficient number of samples with both mutation and gene expression data (>150 samples) from the TCGA data portal [42] through the Xena platform [43].”

(**Methods and Materials**, **Data resources,** Line 400-404)

“It should be noted that we primarily used five common types of cancer as examples to demonstrate the evaluation of the algorithm's performance, including breast invasive carcinoma (BRCA), kidney renal clear cell carcinoma (KIRC), liver cancer (LIHC), glioblastoma (GBM), and stomach adenocarcinoma (STAD). Additionally, we also provided a summary of the performance of PDRWH on the other cancer types.”

**S1 Table. Sample number and mutant gene number in each cancer dataset.**

**S2 Table. Accuracy for the top i-th predicted driver genes by the known general reference driver gene list.**

**S5 Fig. The p-values of personalized drive genes enriched in tumor-specific drive genes on 16 cancer datasets.**

b. Was their proposed method evaluated on genomic and transcriptomic data of patient samples in a pan cancer fashion? Or were the validation experiments run on each cancer type separately? It would be valuable to assess whether their method can successfully identify cancer type-specific drivers within relevant patient populations when evaluated across various cancer types or subtypes. Such a comprehensive pan-cancer evaluation would serve as a definitive proof of concept, demonstrating the method's superiority to predict personalized drivers, specifically tailored to specific patient populations.

**Response:**

Thank you for your question. We apologize for not clearly stating this in the original text. Our method is centered around researching each cancer type separately. We believe that patients with the same driver mutations may share common carcinoma genic mechanisms, a phenomenon that is most pronounced in specific cancer types. Therefore, we aim to enhance personalized driver gene prediction accuracy by utilizing the similarity of expression and mutation profiles between samples within a given cancer type. By focusing on the study of each cancer type, we aim to provide important theoretical support and practical guidance for personalized treatment and precision medicine. This clarification has been added in the revised version.

(**Results, An overview of PDRWH,** Line 94-97)

“We hypothesize that samples with the same cancer types of cancer display higher similarity in molecular characteristics and disease mechanisms compared to samples with different types of cancer. Leveraging samples from the same cancer type, specifically those closely resembling the target sample, has the potential to improve the personalized driver genes prediction.”

c. The study lacks validation on independent datasets such as METABRIC, which houses mutation, copy number and gene expression of 2000 breast cancer patients (<https://www.ncbi.nlm.nih.gov/pmc/articles/PMC3440846/>)

**Response:**

Thank you for providing us with a high-quality dataset. We apologize for not initially specifying that our method falls under unsupervised learning, which may have raised questions about the validation part. Our method belongs to unsupervised learning and does not require model training or validation. We have now included this detail in the main text. This part of the content can help readers understand the characteristics and training methods of the model correctly, thereby gaining a better understanding of the working principles, data requirements, and expected output results of your method. in our future study, we will perform related studies on this dataset. Thanks for your feedback.

(**Discussion** Line 358-362)

“In this study, we have presented an unsupervised learning method to identify patient-specific driver genes by leveraging genome and transcriptome datasets from a cohort. PDRWH, similar to many unsupervised algorithms, can directly uncover hidden patterns and structures in data without the need for explicit model training or a large number of labeled genes, aiming to provide comprehensive support for the analysis of driver genes.”
